# Practical Aspects of Using Large Language Models to Screen Abstracts for Cardiovascular Drug Development: Cross-Sectional Study

**DOI:** 10.2196/64143

**Published:** 2024-09-30

**Authors:** Jay G Ronquillo, Jamie Ye, Donal Gorman, Adina R Lemeshow, Stephen J Watt

**Affiliations:** 1 Worldwide Medical and Safety, Pfizer Research and Development, Pfizer Inc, New York, NY, United States; 2 Pfizer Research and Development UK Ltd, Cambridge, United Kingdom

**Keywords:** biomedical informatics, drug development, cardiology, cardio, LLM, biomedical, drug, cross-sectional study, biomarker, cardiovascular, screening optimization, GPT, large language model, AI, artificial intelligence

## Abstract

Cardiovascular drug development requires synthesizing relevant literature about indications, mechanisms, biomarkers, and outcomes. This short study investigates the performance, cost, and prompt engineering trade-offs of 3 large language models accelerating the literature screening process for cardiovascular drug development applications.

## Introduction

Cardiovascular drug development requires synthesizing information about indications, mechanisms, biomarkers, and outcomes [1,2]. Large language models (LLMs) leveraging billions of data points could accelerate fundamental, resource-intensive aspects of this process, like screening published literature [3]. However, this depends on the design, development, and implementation of LLM instructions (prompt engineering) that work effectively within the context of cardiology [4-6]. To our knowledge, this is one of the first studies investigating LLMs to accelerate the literature screening process for cardiovascular drug development applications [3,4,6,7].

## Methods

### Study Design

Leveraging prior work, a PubMed query using both available Medical Subject Headings (MeSH) and the title and abstract keyword search of MeSH Entry Terms identified observational studies of heart failure that (1) were published from 2013 to 2023, (2) contained at least one relevant biomarker (brain natriuretic peptide, N-terminal pro–atrial natriuretic peptide, N-terminal pro–brain natriuretic peptide, and peak oxygen consumption), and (3) measured long-term outcomes (hospitalization and mortality) [2].

**Figure 1.**
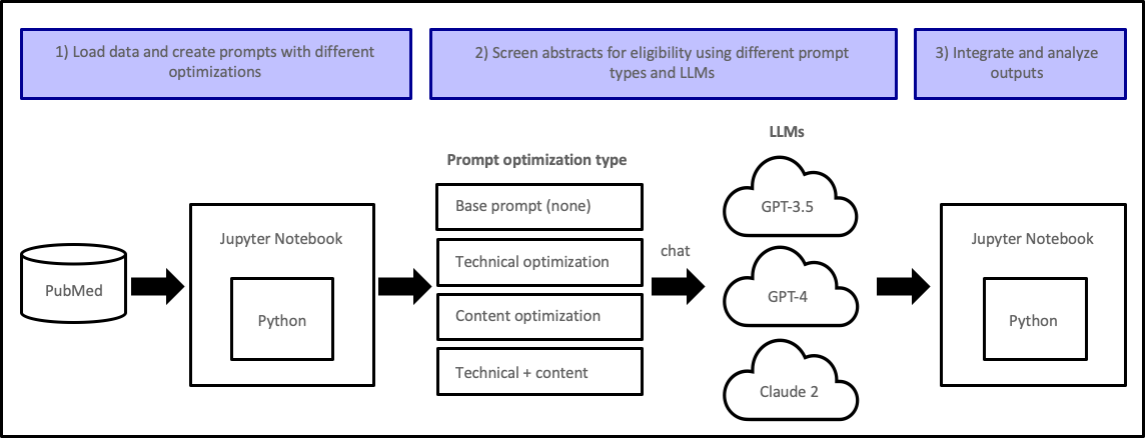
Biomedical informatics pipeline for comparing different LLM and prompt optimization approaches to abstract screening for cardiovascular drug development. LLM: large language model.

Abstracts were extracted through the PubMed application programming interface (API), and LLM instructions (prompts) were created to assess different screening optimization strategies (Figure 1) across LLMs (GPT-3.5 Turbo [OpenAI], GPT-4 [OpenAI], and Claude 2 [Anthropic PBC]) [5]. The “base” LLM prompt (1) presented abstract text, (2) listed two eligibility screening criteria (ie, values found for at least one biomarker and outcome), and (3) instructed LLMs to determine if abstracts met eligibility criteria and return results in a standardized format. “Technical” optimization was defined as adding delimiters to the base prompt delineating key sections (abstract and criteria), while “content” optimization further instructed LLMs to (1) assume a scientific role and (2) address a cardiology drug development target audience [3,5]. The different prompts used in this study are described in Multimedia Appendix 1. Total units of text processed (“tokens”) were estimated using spaCy, and LLM abstract screening costs were estimated using current API prices per million input and output tokens, respectively, for GPT-3.5 (US $0.50 and US $1.50), GPT-4 (US $30 and US $60), and Claude 2 (US $8 and US $24).

A Python script performed data processing and analysis. Accuracy was assessed by comparing LLM outputs against manual epidemiologist review of study suitability for inclusion, with descriptive statistics calculated for each LLM and prompt type. Performance differences between fully optimized prompts (GPT-3.5 vs GPT-4, GPT-3.5 vs Claude 2, and GPT-4 vs Claude 2) were evaluated using the chi-square test. A *P* value of <.05 was considered statistically significant.

### Ethical Considerations

This study did not meet the definition of human participants research and thus did not require institutional review board approval.

## Results

Of 69 articles found in PubMed, 32 (46%) met eligibility criteria after manual review; corresponding LLM screening accuracies are summarized in Table 1. By LLM, the best performances came from the base prompt (GPT-3.5), technical and combined prompts (GPT-4), and technical prompts (Claude 2). Overall, combined prompts for GPT-3.5 and GPT 4 performed similarly against each other (*P*>.99) and against Claude 2 (*P*=.61 against both).

GPT-3.5 processed a total of 124,826 tokens, while GPT-4 and Claude 2 processed 14.4% (N=142,750) and 15.9% (N=144,703) more tokens, respectively. Total costs for GPT-4 (US $4.89) and Claude 2 (US $1.52) were 75.4 and 23.4 times higher, respectively, than total costs for GPT-3.5 (US $0.06).

**Table 1.** Abstract screening accuracies reflecting total abstracts correctly identified by large language models (LLMs) for inclusion and exclusion based on manual review of study suitability, by LLM and prompt optimization type (abstracts: N=69).

Prompt optimization type	Accuracy, n (%)		
	GPT-3.5	GPT-4	Claude 2
Base (none)	43 (62)	40 (58)	35 (51)
Technical	34 (49)	41 (59)	43 (62)
Content	42 (61)	38 (55)	38 (55)
Technical and content	41 (59)	41 (59)	37 (54)

## Discussion

Despite the complex and limited public cardiology data integrated into LLMs, our findings were consistent with similar studies for oncology and current LLM abilities to pass medical licensing exams [4,8]. Performance could be further improved by adding specific examples to the prompt (few-shot prompting) or to the LLM training data (fine-tuning) [4,8,9].

Technical optimizations showed modest performance improvements across some LLMs, indicating one practical way to improve accuracy and prompt readability without significantly expanding the size of input prompts. Standardizing outputs helped generate valid responses, although GPT-4 and Claude 2 still had higher costs as a result of more verbose output. Enterprise LLM–based abstract screening will require balancing prompt performance, cost, and complexity with cardiology subject matter expert capabilities and workflows.

Limitations include a small cardiovascular dataset leveraging proprietary LLMs and only a subset of available optimization techniques. Future efforts must engage diverse scientific communities; develop guardrails to ensure safe and responsible LLM use; and apply data-driven best practices that generalize, optimize, and validate LLM applications and their impact on patients with cardiovascular disease.

## Supplementary material

10.2196/64143 Multimedia Appendix 1 Approach for creating prompts focused on abstract screening for cardiovascular drug development, starting with the base prompt (black) and including content optimization (A) and technical optimization (B-E).

## Abbreviations

API: application programming interface
LLM: large language model
MeSH: Medical Subject Headings
