# Supplementary figures and images for: Practical Aspects of Using Large Language Models to Screen Abstracts for Cardiovascular Drug Development: Cross-Sectional Study
Source: JMIR Med Inform. 2024 Sep 30;12:e64143. doi: 10.2196/64143 (PMC11469161; doi:10.2196/64143)

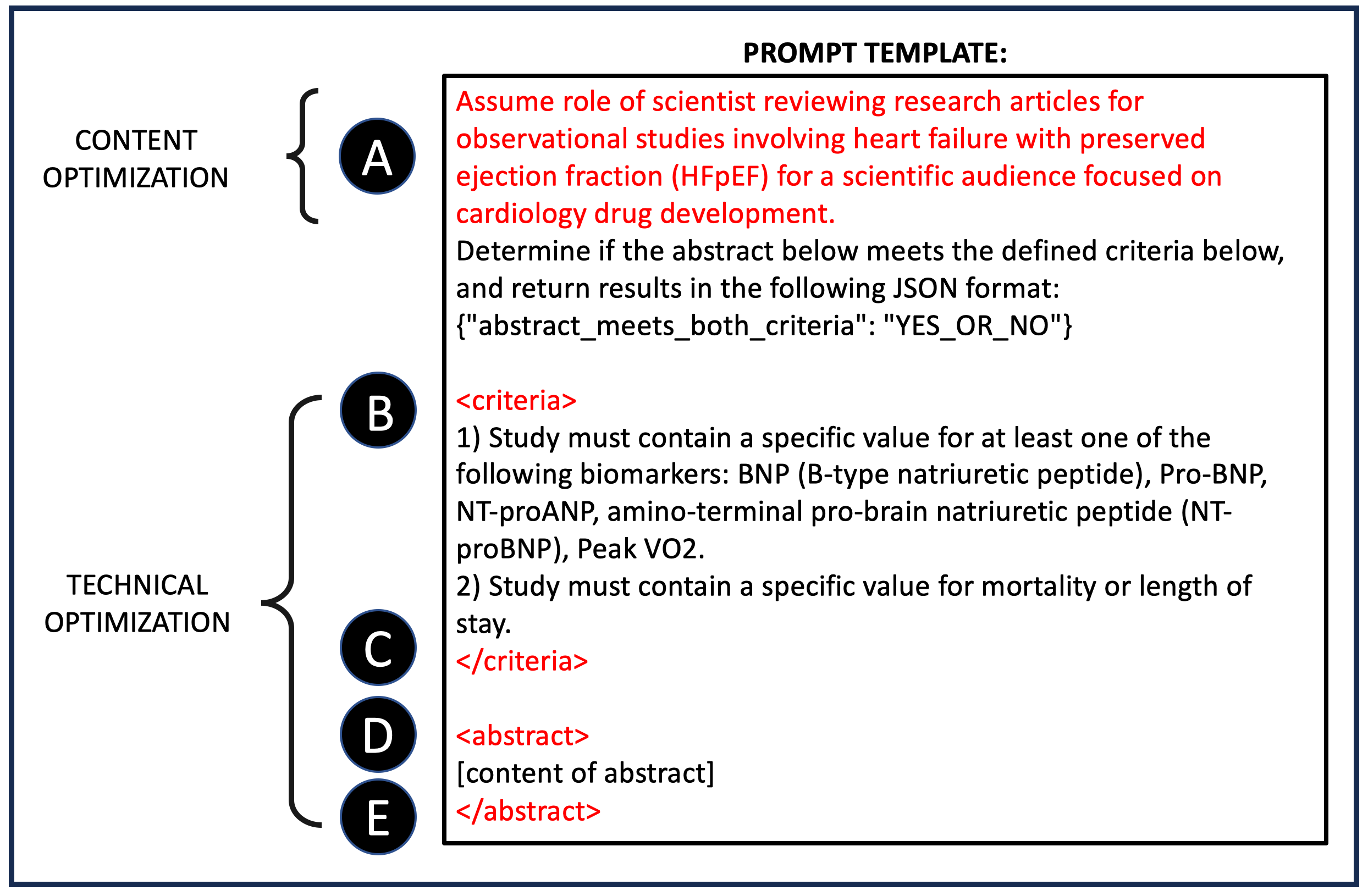

Supplement: Multimedia Appendix 1 [file medinform-v12-e64143-s001.png]
